# Intraoperative low field MRI in transsphenoidal pituitary surgery

**DOI:** 10.1530/EC-18-0140

**Published:** 2018-06-28

**Authors:** Morten Winkler Møller, Marianne Skovsager Andersen, Christian Bonde Pedersen, Bjarne Winther Kristensen, Frantz Rom Poulsen

**Affiliations:** 1 Department of NeurosurgeryOdense University Hospital, Odense C, Denmark; 2 Clinical InstituteUniversity of Southern Denmark, Odense C, Denmark; 3 Department of EndocrinologyOdense University Hospital, Odense C, Denmark; 4 Department of PathologyOdense University Hospital, Odense C, Denmark

**Keywords:** intraoperative MRI, transsphenoidal, microscopic, pituitary, surgery

## Abstract

**Background:**

Intraoperative low field MRI (iMRI, 0.15 T) during transsphenoidal surgery on pituitary adenomas (PAs) may significantly improve tumor removal. However, extensive surgery can lead to pituitary hormone deficiency. Furthermore, introduction of iMRI will prolong duration of surgery, which may elevate risk of postoperative infections.

**Methods:**

Overall, 180 transsphenoidal surgeries for PAs from 2007 to 2015 were included. IMRI was available from 2011 to 2015, during this period 67/78 (86%) surgeries were with iMRI (iMRI, *n* = 67). A total of 113 surgeries were performed without iMRI (controls). All surgical procedures were performed by microscopic technique. Tumor size, hormonal status and vision were assessed before surgery and 3–5 months postoperatively.

**Results:**

Gross total resection (GTR), mean tumor remnant volume and ∆-volumes were comparable between iMRI and controls: 15% (10/66) vs 23% (26/109) (*P* = 0.17), 2.97 cm^3^ (0.9–5) vs 2.1 cm^3^ (1.6–2.6) (*P* = 0.3) and 4.5 cm^3^ (3.6–5.5) vs 5.1 cm^3^ (4.2–6) (*P* = 0.4), respectively. Duration of surgery was significantly longer during iMRI vs controls: 126 min (117–135) vs 98 min (92–103) (*P* < 0.001). New pituitary–adrenal deficiency in iMRI vs controls was seen in 35% (17/48) and 35% (23/66) of surgeries, respectively (*P* = 0.95). New thyroid deficiency was found in 33% (13/29) and 41% (28/69) and visual field deficiencies improved in 44% (19/43) and 38% (23/60) in iMRI vs controls, respectively (*P* > 0.1).

**Conclusion:**

Tumor remnant after pituitary surgery was not significantly reduced using intraoperative low field MRI. Duration of surgery was increased in iMRI, but was not associated with increased infection rate. Pituitary hormonal function and vision were comparable between iMRI and controls.

## Introduction

Pituitary adenomas (PAs) account for 10–25% of intracranial tumors (1, 2, 3). They arise from adenohypophyseal cells, despite their benign nature, 25–55% of PAs show invasive growth (4), expanding into suprasellar (5), infrasellar and/or parasellar regions (6). Pressure on neighboring structures, including anterior pituitary cells, optic chiasma and adjacent nerves in cavernous sinus may cause hypopituitarism (7), vision field impairment and/or ophthalmoplegia (8).

For other PAs, except prolactinomas, surgery is the first choice of treatment. The primary surgical technique has been transsphenoidal surgery since Hardy introduced surgical microscopes in 1962 (9). Further development of this technique has improved gross total resection rate (GTR) (10). Intraoperative low field MRI (iMRI) has been proven useful for visualizing tumor remnant during surgery (11). iMRI may improve and facilitate GTR (leaving no adenoma remnant on postoperative MRI) as iMRI has been used, successfully, for other intracerebral tumors, such as gliomas (12).

In general, surgical risk includes postoperative anterior hypopituitarism or impaired vision, especially in macroadenomas (≥10 mm). Additionally, rhinoliquorrhea and diabetes insipidus (DI) are common complications (13, 14).

PAs are classified into clinically non-functioning pituitary adenomas (NFPAs) to clinical prolactinoma (PRL)-, adrenocorticotropic hormone (ACTH)-, growth hormone (GH)- or thyroid-stimulating hormone (TSH)-secreting adenomas (15).

Previous studies reported that iMRI augmented adequate resection of PAs (16, 17), but no studies have investigated whether presumed increased resection using iMRI is correlated with postoperative pituitary function and vision improvement.

The purpose of this retrospective study was to determine whether low field iMRI during transsphenoidal pituitary surgery significantly (1) improved tumor resection, (2) augmented pituitary hormone deficiency and/or (3) influenced (post)operative complications.

## Methods

### Study design

In this retrospective cohort study, the inclusion criteria were transsphenoidal surgery for PA from 2007 to 2015. Patients were identified using the Patient Registry and ICD-10 diagnostic code D352 combined with surgical code AAE10. A low field iMRI (Medtronic, Polestar 0.15 T) was installed at the department of neurosurgery in the fall of 2011 and used for all cases of transsphenoidal surgery, except in a few cases of scanner malfunction or where patient stature were incompatible with the scanner. Patients were stratified into two patient cohorts operated with and without (control group) use of iMRI (iMRI). All surgical procedures were performed by the same two neurosurgeons. Both surgeons were experienced and performed the procedures routinely throughout this period.

### Surgical technique

After induction of general anesthesia, a standard microscope (Zeiss, Pentoro) and neuronavigation (Medtronic, Minneapolis, MN, USA)-guided submucosal paraseptal transsphenoidal microsurgical technique was used in all patients. Adenomas were removed using blunt currettes. After adenoma removal, hemostasis was achieved with temporary placement of Spongostan and, if necessary, Surgiflo or FlowSeal. Dura was closed using Tachoseal and in some cases the sellar floor was reconstructed using titanium mesh or septal bone. The nasal septum was repositioned and fixed with nasal packing for 12–24 h. Lumbar drainage was not used routinely.

In the iMRI group, the patient’s head was fixed in a dedicated titanium head holder with MR coil (Medtronic). A preoperative T1 iMRI scan without contrast was performed immediately prior to surgery and at least one iMRI scan was performed during surgery (T1 with intravenous contrast, Dotarem). The first iMRI was performed when resection was considered adequate by the surgeon. If the scan showed accessible tumor remnant, an additional attempt to remove tumor was made. All patients were admitted to a semi-intensive neurosurgical ward for postoperative monitoring. If there were no perioperative surgical complications, patients were routinely transferred to the endocrinology ward the following day.

### Data collection

#### Magnetic resonance imaging

Knosp (6) and Hardy (5) classifications were used to classify adenoma extension based on the patients preoperative MRI (1.5 or 3 T). Tumor volume was calculated using 3D volumetric analysis in Horos on basis of axial, frontal or sagittal sections on T1-weighted images with contrast. Similarly, tumor remnant volume was calculated on routine follow-up MRI scans performed 4–6 months after surgery. Duration of surgery and complications within 30 days were registered. All image analysis was performed blinded to the observer with respect to usage of iMRI.

Assessment of the preoperative and postoperative MRIs was performed blinded to the observer concerning the use of iMRI, pituitary function and vision impairment. Cases with uncertainty concerning tumor delineation were discussed with senior neurosurgeons.

#### Pituitary function

Pituitary hormone evaluation was assessed preoperatively and postoperatively based on clinical biochemical data including the hypothalamic–pituitary–somatotropic axis (s-GH and s-IGF-1), hypothalamic–pituitary–gonadal (HPG) axis (s-FSH, s-LH, s-estradiol, s-total testosterone and s-sexual hormone-binding globulin (s-SHBG)), hypothalamic–pituitary–thyroid (HPT) axis (s-TSH, s-T4 and s-thyroid-binding globulin (s-TBG)) and hypothalamic–pituitary–adrenal (HPA) axis (s-ACTH, s-cortisol and Synacthen test). Deficiency in either axis was defined as biochemical data outside reference values for the specific hormone or if patients were already on substitution therapy. Intact function was defined as hormone levels within the normal range, whereas increased values for IGF-1, cortisol or T4 lead to specific tests for secreting adenomas. After initial screening for a prolactinoma, the adenoma was clinically divided into NFPA, ACTH-secreting adenomas (hypercortisolism defined by elevated 24-h urinary free cortisol, non-suppressability of cortisol after 1 mg of dexamethasone overnight to >50 nmol/L and if required inferior petrosal sinus sampling to localize the PA or a GH-secreting adenoma (no suppression of GH to <1 µg/L after an OGTT and elevated IGF-I). The exact diagnosis was based on the postoperative pathological and immunohistochemical analysis.

After surgery, the patients were monitored for development of DI. Postoperative pituitary function was assessed 6 weeks after surgery.

### Vision

Vision was evaluated in all patients able to cooperate. Visual field was evaluated by quadrant affected (upper-lower temporal/upper-lower nasal). This was combined with mean deviation (MD) measured in decibel (dB) for each eye, both pre- and postoperatively. Central vision deficiency was evaluated by rapidly declined vision by Snellens board or blindness. Based on MD, vision was categorized as intact vision, field vision deficiency or central vision impairment or combinations thereof.

### Statistical analysis

All data were entered into a REDcap database (a secure web application for building and managing online surveys and databases) (18), with assistance from OPEN (OPEN Odense Patient Data Explorative Network) and analyzed using Student’s *t*-test and chi-squared test (STATA/IC 15.0).

### Ethics

The study was approved by the Danish Patient Safety Authority (ID 3-3013-1765/1/) and the Danish Data Protection Agency (ID 16/25477). The Danish Patient Safety Authority allowed us to use patient journal information without the consent of each patient. It was approved on 8/7/2016, with case number: 3-3013-1765/1/.

## Results

### Patient inclusion

**Figure 1:**
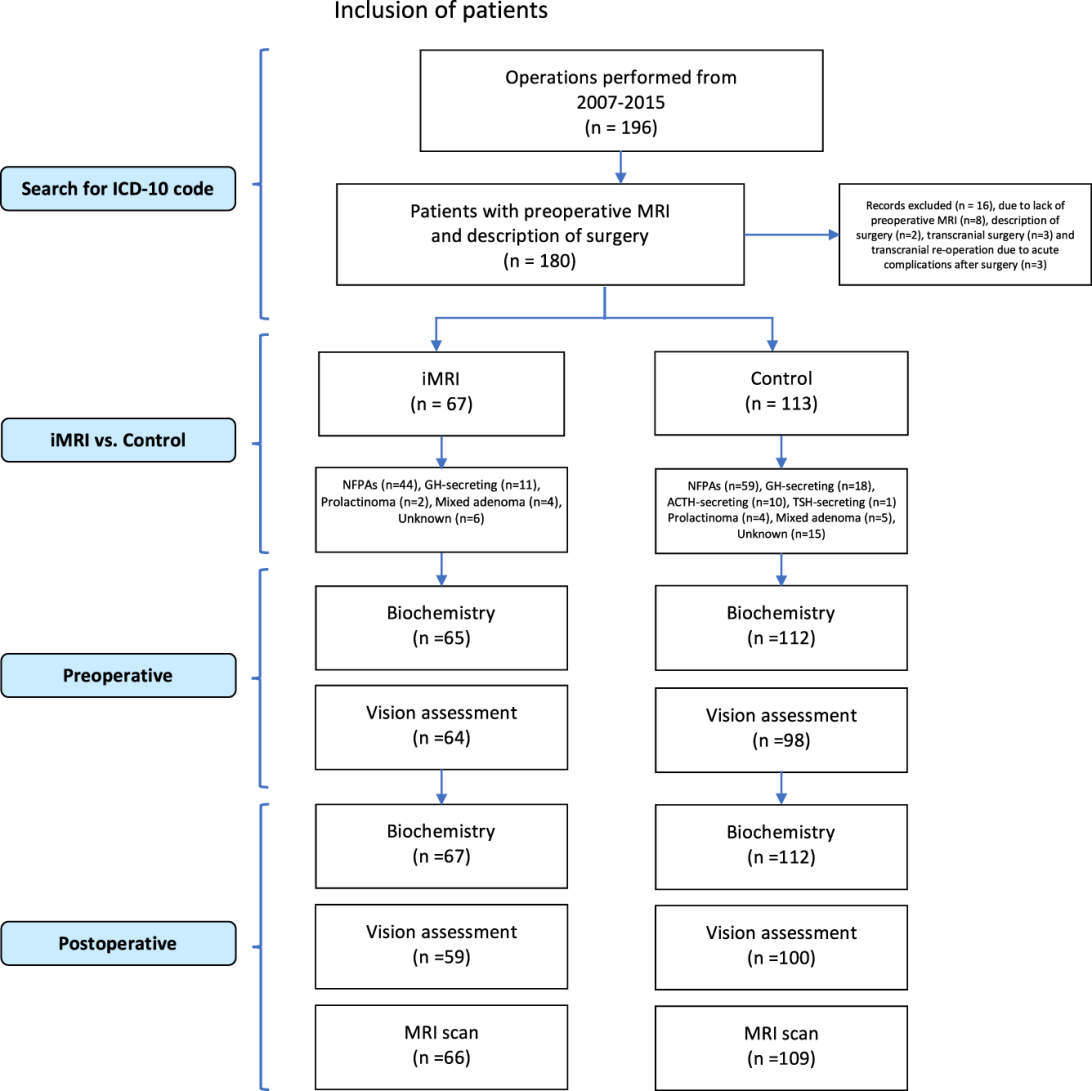
PRISMA diagram.

196 operations were performed during 2007–2015. Sixteen cases were excluded since either no preoperative MRI scan in (8/16) or in (2/16) cases no surgical description was available (Fig. 1, PRISM diagram). Three out of 16 were primarily operated with a transcranial approach due to extensive suprasellar expansion of the adenoma. The last three cases had acute transcranial surgery during the immediate postoperative period due to expanding intrasellar hematoma or excessive rhinoliquorrhea. During 2011–2015, 86% (67/78) surgeries were performed using iMRI (iMRI, *n* = 67). During the time period 2007–2015, 113 patients were operated without the use of iMRI leaving a total of 180 patients included in this study (Fig. 1; Table 1).

**Table 1:** Characteristics.

Characteristics	
*n*	180
Gender	
Male/female	96/84
Median age at surgery (years) (range)	58 (17–87)
Primary operation	161 (89.4)
Re-operation	16 (8.9)
Multiple re-operation	3 (1.7)*
Pituitary function	
HPA axis	
Intact	115 (63.9)
Deficient	47 (26.1)
Unknown	8 (4.5)
Cushing’s	10 (5.6)
HPT axis	
Intact	109 (60.6)
Deficient	60 (33.3)
Unknown	7 (3.9)
Elevated T4	4 (2.2)
HPG axis	
Intact	75 (41.7)
Deficient	95 (52.8)
Unknown	10 (5.5)
ADH status	
Intact	176 (97.7)
Deficient	4 (2.3)
Axes deficient (%)	
0	53 (29.4)
1	42 (23.3)
2	29 (16.1)
3	27 (15)
Unknown	29 (16.1)
Type of pituitary adenoma	
NFPA	103 (57.2)
GH secreting	29 (16.1)
ACTH secreting	10 (5.6)
TSH secreting	1 (0.6)
Prolactinoma	6 (3.3)
Mixed adenoma	9 (5)
Other anomaly in sella	11 (6.1)
Pituitary apoplexy	7 (3.7)*
Unknown	11 (6.1)
Vision	
None	60/50
Field	103/107
Central	13/15

*Two patients had three surgical procedures and a single patient underwent five operations. Pituitary apoplexia were found among the other adenomas.

### Resection

**Figure 2:**
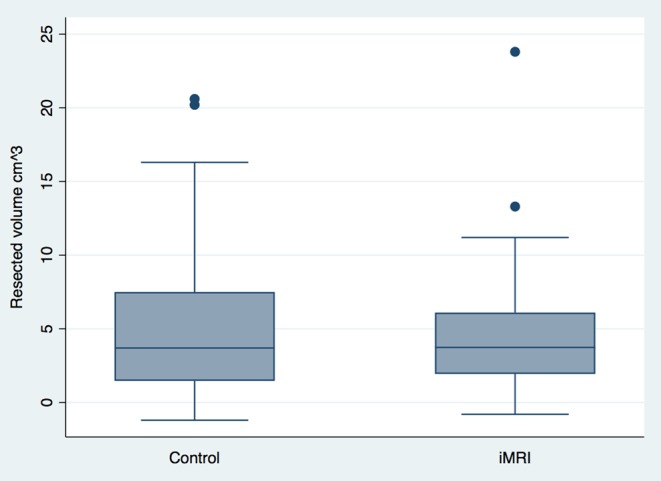
∆-volume box-plot.

GTR was achieved in 15% (10/66) in the iMRI group vs 23% (25/109) in the control group, *P* = 0.26, chi-squared. The mean tumor remnant volume on the postoperative MRI was 2.97 cm^3^ (0.9–5) in the iMRI group vs 2.1 cm^3^ (1.6–2.6) in the control group. The mean resected tumor volume (∆-volume) was 4.5 cm^3^ (3.6–5.5) vs 5.1 cm^3^ (4.2–6), respectively (Fig. 2 and Table 2).

**Table 2:** Tumor volume, resection and complications.

	iMRI (*n* = 66)	Control (*n* = 109)	*P* value
Preoperative (cm^3^) (95% CI)	7.5 (5.32–9.58)	7.16 (6.02–8.31)	0.8
No tumor remnant, *n* (%)	10 (15)	26 (23)	0.26
Tumor remnant	56 (85)	83 (77)	0.17
Postoperative (cm^3^) (95% CI)	2.97 (0.94–5.01)	2.09 (1.58–2.6)	0.3
∆-Volume (cm^3^) (95% CI)	4.52 (3.56–5.49)	5.11 (4.2–6.03)	0.4
Duration of surgery (mean in min) (95% CI)	127 (118–136)	96 (91–101)	**<0.0001**
Complications after 30 days	21 (31.8)	35 (30.7)	0.87
Deep venous thrombosis (DVT)	0	0	
Lung emboli (*n*) (%)	2 (2.9)	3 (2.5)	0.86
Rhinoliquorrhea (*n*) (%)	10 (14.7)	11 (11.7)	0.55
Meningitis (*n*) (%)	3 (4.4)	3 (3.3)	0.71
Hematoma	0	2 (1.7)	
Hemorrhage	0	2 (1.7)	
Apoplexy	0	0	
Other	10 (16.2)	20 (20)	0.52
Lumbar drain	4	4	
Pneumo- or hydrocephalus with external cerebral drain	1	4	
CNS infection	3	3	
Liquorfistel operation	2	3	
SIADH after surgery	7 (10.8)	3 (2.7)	**0.026**

Adenoma extension was classified by Knosp and Hardy classifications as shown in the Supplementary Table 1 (see section on supplementary data given at the end of this article). Adenomas were grouped by the Knosp classification into 0–2 and 3–4. Comparison between the iMRI group and the control group showed no difference in the amount of (GTR), mean remnant volume or ∆-volume (Table 3).

**Table 3:** Tumor volume by Knosp classification.

	iMRI	Control	*P* value
	Right/left	Right/left	Right/left
Pre op volume			
0–2 (cm^3^)	5.2/5.7	5.1/5.2	0.84/0.50
3–4 (cm^3^)	14.0/12.1	12.2/12.7	0.56/0.86
GTR			
0–2 (*n* = 125) (%)	9 (18.4)/9 (18.4)	19 (25.0)/22 (27.9)	0.39/0.25
3–4 (*n* = 49) (%)	1 (5.9)/1 (5.9)	6 (18.8)/3 (10.3)	0.22/0.57
Remnant volume			
0–2 (cm^3^)	1.6/1.9	1.4/1.4	0.63/0.27
3–4 (cm^3^)	6.9/5.8	3.7/3.9	0.26/0.50
∆-Volume			
0–2 (cm^3^)	3.7/3.9	3.7/3.8	0.90/0.95
3–4 (cm^3^)	7.0/6.3	8.5/8.7	0.39/0.14

0–2: Knosp classification 0, 1 or 2. 3–4: Knosp classification 3a, 3b or 4.

The duration of surgery was significantly longer in the iMRI group (126 min range: 117–135) compared to the control group (98 min range: 92–103), *P* value <0.001. However, there was no difference in the rate of complications 30 days postoperatively between the two groups. Complications included lung embolism, meningitis or rhinoliquorrhea (Table 2).

### Pituitary function

**Figure 3 fig3:**
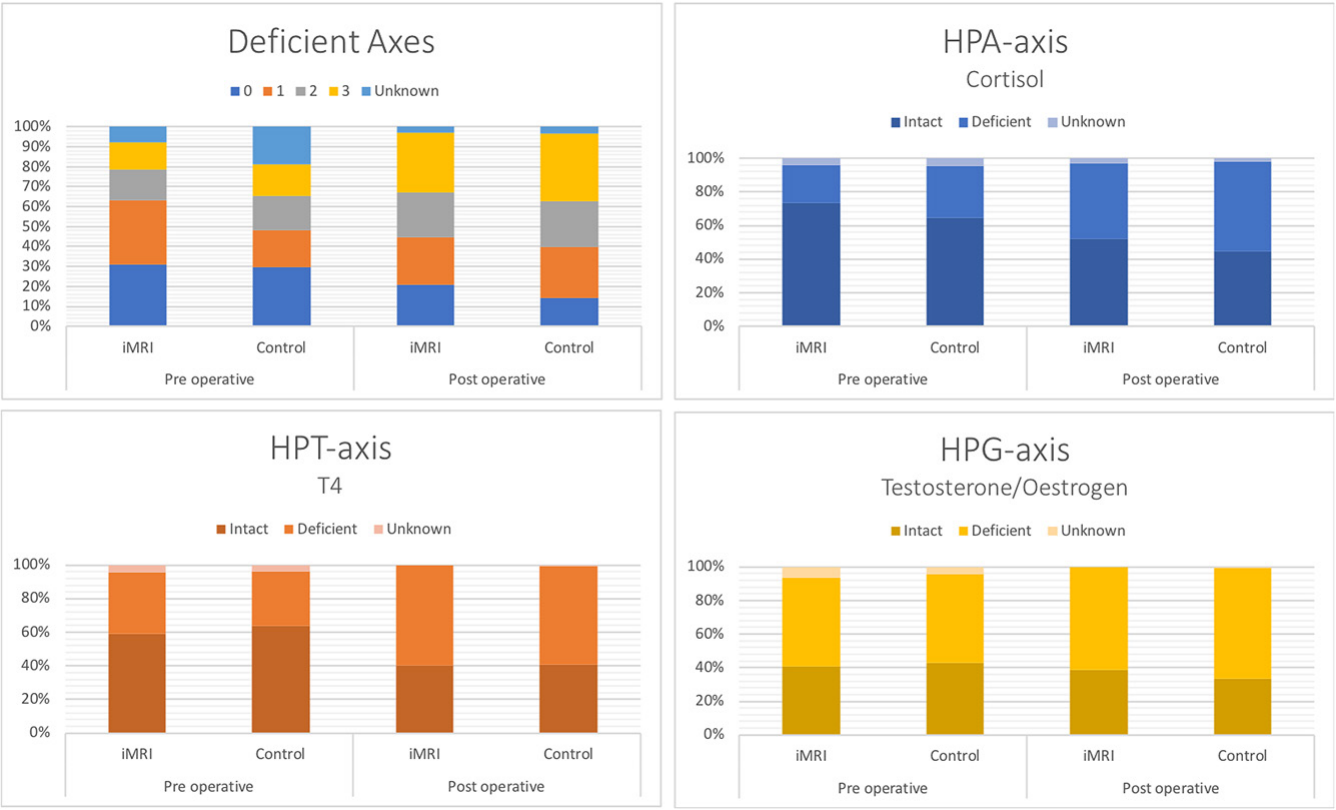
Deficient axes and specific axis function.

HPA axis was intact in 115 cases preoperatively. Of these, 63% (31/48) of the iMRI group remained intact after surgery vs 65% (43/66) in the control group, *P* = 0.95. In 15% (7/47) of the cases preoperative lost function was regained after surgery; 20% (3/15) in the iMRI group vs 12.5% (4/32) in the control group (Fig. 3). HPT axis was intact in 108 cases preoperatively, whereas postoperatively 66% (26/39) patients of the iMRI group vs 59% (43/69) of the controls were intact in this axis, *P* = 0.46 (Table 4). One case in the control group regained normal thyroid function after surgery.

**Table 4:** Postoperative pituitary function.

	iMRI (*n* = 67)		Control (*n* = 113)		Total
HPA axis (%)					
Intact	35 (52)		50 (44.3)		85 (47.2)
Deficient	30 (44.8)		60 (53.1)		90 (50)
Unknown	2 (3)		2 (1.8)		4 (2.2)
Increase	0		1 (0.9)		1 (0.6)
HPT axis (%)					
Intact	27 (40.3)		45 (39.8)		72 (40)
Deficient	40 (59.7)		65 (57.5)		105 (58.4)
Increase	0		2 (1.7)		2 (1.1)
Unknown	0		1 (0.9)		1 (0.6)
HPG axis (%)					
Intact	26 (38.8)		38 (33.6)		64 (35.6)
Deficient	41 (61.2)		74 (65.5)		115 (65)
Unknown	0		1 (0.9)		1 (0.6)
ADH function (%)					
Intact	55 (82.1)		83 (73.5)		138 (76.7)
Deficient	12 (17.9)		26 (23)		38 (21.1)
Unknown	0		4 (3.6)		4 (2.3)
Deficient axes (%)					
0	14 (20.9)		16 (14.2)		30 (16.7)
1	16 (23.9)		29 (25.7)		45 (25)
2	15 (22.4)		26 (23)		41 (22.8)
3	20 (29.9)		38 (33.6)		58 (32.2)
Unknown	2 (3)		4 (3.5)		6 (3.3)
	**iMRI**		**Control**		***P* values**
	Intact	Deficient	Intact	Deficient	
Post op control					
HPA axis, pre op intact *n* = 114 (%)	31 (63.3)	17 (34.7)	43 (65.2)	23 (34.8)	0.95
HPT axis, pre op intact *n* = 108 (%)	26 (66.7)	13 (33.3)	41 (59.4)	28 (40.6)	0.46
Fertile women, menstrual status pre op, *n* = 30	6 (66.7)	3* (33.3)	11 (52.4)	10 (47.6)	0.47
If intact pre op, *n* = 16	4 (80)	1 (20)	9 (81.8)	2 (18.2)	0.93
If deficient pre op, *n* = 15	2 (40)	3* (60)	2 (20)	8 (80)	0.41
Testosterone level pre op, *n* = 72	8 (33.3)	16 (66.7)	19 (39.6)	29 (60.4)	0.61
Intact, *n* = 33	6 (75)	4 (25)	13 (68.4)	10 (34.5)	0.85
Deficient, 39	2 (25)	12 (75)	6 (31.6)	19 (65.5)	0.47
	Secreting	Non-secreting	Secreting	Non-secreting	
GH-secreting adenomas, *n* = 29	7 (63.6)	4 (36.4)	9 (50)	9 (50)	0.70

*1 Female with Hormonspiral.

Patients who were intact in both HPA and HPT axes postoperatively showed a surgical reduction in adenoma volume by 3.8 cm^3^ (95% CI: 2.8–4.9), while in those with deficient function in both these axes had adenoma volume reduced by 4.8 cm^3^ (95% CI: 3.6–5.1), *P*-value = 0.22.

### HPG axis

The cohorts consisted of 32 males and 35 females in the iMRI group vs 64 males and 49 females in the controls group. 26% (9/35) patients in the iMRI group were fertile women vs 47% (21/49) in the control group. In the iMRI group 67% (6/9) women had regular menstrual cycles vs 52% (11/21) in the control group, and 33% (3/9) had irregular menstrual cycles in the iMRI group vs 48% (10/21) in the control group postoperatively, *P* = 0.93. Table 4 shows the distribution of menstruation status before and after surgery. There were no differences between the groups.[Fig fig3]

The males were divided into groups based on their testosterone level. This shows that 42% (10/24) males in the iMRI group had normal testosterone levels postoperatively, compared to 48% (23/48) in the control group.

In summary, there were no statistical significant difference in postoperative pituitary function between the iMRI group and the control group.

### GH-secreting adenomas

Eleven in the iMRI group vs 18 in the control group were operated due to a GH-secreting adenoma. 9% (1/11) in the iMRI group were microadenomas vs 17% (3/18) in the control group. All of them had increased GH and IGF-1 values preoperatively and lack of suppression of GH during OGTT.

Six weeks after surgery, 36% (4/11) in the iMRI group had normal IGF-1 values and sufficient GH suppression during OGTT compared to 50% (9/18) in the control group, *P* = 0.70 (Table 4).

### Diabetes insipidus

Four patients had DI preoperatively. In one patient, this disappeared after surgery. Overall, 18% (12/67) patients required postsurgical desmopressin treatment permanently in the iMRI group vs 23% (26/113) in the control group. Transient postoperative SIADH was found in 11% (7/67) in the iMRI group vs 2.7% (3/109) in the control group, a significant difference, *P* = 0.026.

### Vision

In the iMRI group, 32% (19/59) patients showed vision improvement, ranging from improvement in presurgical visual field deficiencies to intact vision, postoperatively compared to 31% (30/98) in the control group. 5% (3/59) of cases experienced worsened vision, ranging from visual field deficiencies to central vision defects, postoperatively in the iMRI group compared to 4% (4/99) cases in the control group. Among cases with preoperative central vision impairment, 67% (2/3) patients showed improvements in the iMRI group compared to 70% (7/10) in the control group.

Stratifying change in vision according to the Hardy classification grades B and C (data shown in Table 5), also revealed no statistically significant differences between iMRI and controls (*P* = 0.2 and *P* = 0.65).

**Table 5:** Vision by Hardy classification.

Suprasellar expansion	iMRI	Control	*P* values
**Overall**	**(*n* = 64)**	**(*n* = 99)**	
Right eye			
Impaired field pre (%)	43 (67.2)	60 (60.6)	
Improved	19 (44.2)	23 (38.3)	0.55
Unchanged	21 (48.8)	34 (56.7)	0.43
Worsened	1 (1.7)	0	NA
Intact pre	21 (32.8)	39 (39.4)	
Unchanged	16 (76.2)	30 (76.9)	0.98
Worsened	2 (9.5)	4 (10.3)	0.93
Impaired central pre	3 (4.7)	10 (9.9)	
Improved	2 (66.6)	7 (70)	0.96
Left eye			
Impaired field pre (%)	45 (70.3)	62 (62.6)	
Improved	16 (35.6)	17 (27.4)	0.37
Unchanged	26 (57.8)	42 (67.7)	0.29
Worsened	1 (1.8)	1 (1)	0.82
Intact pre	18 (28.1)	32 (32.3)	
Unchanged	14 (77.8)	26 (81.3)	0.92
Worsened	1 (5.6)	2 (6.3)	0.92
Impaired central pre	3 (4.6)	12 (12.1)	
Improved	2 (66.6)	7 (58.3)	0.9
**Hardy grade B**	**(*n* = 28)**	**(*n* = 45)**	
Right eye			
Impaired field pre (%)	22 (81.5)	30 (66.7)	
Improved	12 (54.6)	11 (36.7)	0.2
Unchanged	8 (36.4)	18 (60)	0.09
Worsened	0	1 (2.2)	NA
Intact pre	5 (17.9)	15 (33.3)	
Unchanged	5 (100)	12 (80)	0.76
Worsened	0	0	
Impaired central pre	2 (7.4)	4 (8.9)	
Improved	1 (50)	3 (75)	0.78
Left eye			
Impaired field pre (%)	23 (82.1)	33 (73.3)	
Improved	10 (43.5)	9 (27.3)	0.21
Unchanged	11 (47.8)	22 (66.7)	0.16
Worsened	1 (4.3)	1 (2.2)	0.8
Intact pre	4 (14.3)	11 (24.4)	
Unchanged	4 (100)	10 (90.9)	0.91
Worsened	0	0	
Impaired central pre	3 (10.7)	3 (6.7)	
Improved	0	0	NA
**Hardy grade C**	**(*n* = 18)**	**(*n* = 22)**	
Right eye			
Impaired field pre (%)	14 (77.8)	19 (86.4)	
Improved	4 (28.6)	7 (36.8)	0.61
Unchanged	10 (71.4)	10 (52.6)	0.27
Worsened	1 (5.6)	0	NA
Intact pre	3 (16.7)	2 (9.1)	
Unchanged	2 (66.7)	2 (100)	0.76
Worsened	1 (33.3)	0	NA
Impaired central pre	1 (5.6)	5 (22.7)	
Improved	1 (100)	3 (60)	0.75
Left eye			
Impaired field pre	16 (88.9)	20 (90.9)	
Improved	3 (18.8)	5 (25)	0.65
Unchanged	13 (81.3)	14 (70)	0.5
Worsened	0	0	
Intact pre	0	0	
Impaired central pre	0	7 (31.8)	
Improved	0	4 (57.1)	NA

## Discussion

### Surgery

In the present study, we found no significant differences between use and non-use of low field iMRI during surgery for PAs. Our results were in contrast to previous findings, where the use of low field iMRI increased the likelihood of GTR by 3–33% (19). The lack of effect of low field iMRI on GTR in the present study could be due to (1) the image quality and resolution of a low field iMRI (0.15 T) not being adequate for evaluating tumor remnant after pituitary surgery, (2) an identified remnant on iMRI, does not necessarily make it possible to remove it without undue risk for the patient or (3) the interpretation of postoperative MRI was performed by neurosurgical personnel and not by a neuroradiologist.

Although surgery was performed by the same two neurosurgeons, the relatively low number of patients without PA remnant on the first postoperative control MRI may reflect the relatively low case volume. In contrast to our findings, this should, however, argue for a positive effect of the iMRI technique. Today the case volume is higher and the endoscopic surgical technique was introduced in January 2016 with both surgeons operating in collaboration. These results are being analyzed and compared to the traditional method described in this study.

Duration of surgery was prolonged to 126 min (range: 117–135) in the iMRI group compared to the control group (98 min, range: 92–103), *P*-value <0.001. This is comparable with previous findings (11, 20, 21).

The largest study on 229 PAs applying iMRI in pituitary surgery (16) reported that 20.5% (47/229) cases had a tumor remnant, which was eligible for further resection. In contrast to the present study, the 229 patients were selected for the use of iMRI due to large tumors, with extensive suprasellar or retro/parasellar expansion and no controls were included (16). In addition, no volumetric analysis of the tumor volume preoperatively or postoperatively was performed (16). Hence, the selection of study participants and lack of data on the size of tumor volume may have influenced the results.

In a review paper on 0.15 T iMRI (20), no increase in GTR was described in two out of five studies. Our study showed an 18.4% GTR in Knosp grade 0–2 and a 5.9% GTR in Knosp grade 3–4 cases, with no statistically significant difference between iMRI and controls. Better resection of less invasive adenomas by the use of iMRI is, however, supported by findings in previous studies (16, 22, 23).

Transsphenoidal surgery is considered relatively safe, and accordingly, we did not experience severe complications in this study. However, duration of surgery in the iMRI group was increased by approximately 30 min compared to the controls (*P* value <0.001). The longer duration did not result in any significant differences in the rate of complications between the groups, even when considering the three excluded cases, having transcranial surgery due to postoperative acute complications. This is also comparable with a previous study (19).

Because of introduction of new equipment, the iMRI, a learning curve might have been expected during the first year. However, there was no statistically significant difference in duration of surgery, tumor remnant volume and complication rates when comparing surgeries performed during the first year of iMRI and later (data not shown).

### Pituitary function

There was no difference in postoperative pituitary function with or without the use of iMRI. 35% (17/48) in the iMRI group vs 38% (23/63) in the control group developed HPA-axis deficiency. Similarly, 33% (13/39) developed HPT-axis deficiency in the iMRI group vs 41% (28/69) in the control group. These findings are similar to a previous study (11). Postoperative HPA-axis deficiency is concluded based on an insufficient response to a Synacthen test (24) while central hypothyroidism is based on low T4 combined with a (sub)normal TSH (25). In a study by Berkmann *et al.* (17), an insignificant difference was found between iMRI and controls in both HPA (15% vs 13%) and HPT (18% vs 13%) axes, respectively.

Overall, 15% (7/47) of the cases showed remission of HPA-axis function, comparable to previously published rates of 16–41% (7, 26); however, their results were only based on NFPAs. HPT-axis remission was lower in our study where only 1.7% (1/60) cases regained function compared to previous studies reporting that 7–14% (7, 26) of the patients normalized function. Postoperatively, 18% (12/67) in the iMRI group presented with DI, compared to 23% (26/113) in the control group. This was slightly higher than previous published studies (0.5–15%) (13, 14).

Interestingly, there was a tendency for patients with both intact HPA and HPT axes postoperatively to have a lower surgical reduction in adenoma volume. Although not statistically significant, this suggests that higher degree of surgical resection is associated with a higher risk of postoperative pituitary insufficiency. Further studies are needed to clarify this.

We found no effect of using low field iMRI regarding GH-secreting adenomas (*P* = 0.70). Remission of GH secretion was 45% (13/29), which showed that even a small tumor remnant not visualized on 0.15 T iMRI was capable of GH hypersecretion. In contrast to our findings, iMRI was found favorable for complete resection of GH-secreting adenomas in a previous study (27).

In total, 14% (4/29) of the GH-secreting adenomas were microadenomas. However, in our experience, low field iMRI has a limited role for microadenomas surgery, since GH-microadenomas are too small to be visualized using a 0.15 T iMRI.

### Vision

In the present study, 32% (19/59) cases in the iMRI group showed improvement of preoperative deficiency in field of vision. This is less than reported in a recent study on 47 patients by Luomaranta *et al*. (28) where a 50% remission to normal vision was found. However, in Luomarantas study, three patients were excluded due to deterioration of vision after surgery. Our study showed worsened vision in 7 of 165 (4%) patients who were able to participate in the assessment of field vision deficiencies. These issues considered, the risk of impaired vision after PA surgery seems to be consistent throughout the literature and comparable to the results of the present study. This is further substantiated by a study by Wu *et al*. (20), where they found worsened vision in 3% of cases after surgery.

### Limitations and strengths

This study evaluated the effects of the use of iMRI in transsphenoidal operations on PAs. Patients were selected by surgical procedure only. A few cases were discarded due to lack of availability of both a preoperative MRI scan or an available description of the surgical procedure. These inclusion criteria resulted in a highly diverse but very representative group of patients with PAs. Furthermore, all patients were operated by the same two surgeons.

## Conclusion

Use of intraoperative low field MRI in transsphenoidal pituitary surgery was not associated with increased GTR or reduced volume of tumor remnants. The duration of surgery was prolonged when iMRI was used, but it was not associated with increased infection rates. Changes in postoperative pituitary function and visual fields were comparable.

## Supplementary Material

Supporting Table 1

## Declaration of interest

The authors declare that there is no conflict of interest that could be perceived as prejudicing the impartiality of the research reported.

## Funding

This study was funded by Lundbeck Foundation, through the Danish Neurosurgical Society (DNKS).
