# Supplementary material for: Intraoperative low field MRI in transsphenoidal pituitary surgery
Source: Endocr Connect. 2018 Jun 28;7(7):897–906. doi: 10.1530/EC-18-0140 (PMC6063879; doi:10.1530/EC-18-0140)
Supplement: Supporting Table 1 [file ec-7-897-t001.pdf]

Appendix – Knosp and Hardy

Hardy classification	iMRI, n (cm^3) (n=67)	Control, n (cm^3) (n=113)	Total, n (cm^3) (n=180)	
A	19 (4.1)	34 (2.5)	53	P=0.065
B	28 (7.5)	45 (7)	73	P=0.76
C	18 (10.5)	22 (12.1)	40	P=0.33
D	1 (15.7)	6 (17.3)	7	
E	1 (8.2)	2 (13.6)	3	

Distribution of patients by Hardy Classification

Knosp classification	iMRI (n=67)	Control (n=113)	Total (n=180)
	Right/left, n	Right/left, n	Right/left, n
0	8/11	16/22	24/33
1	23/23	34/29	57/52
2	19/15	30/32	49/47
3a	12/4	21/10	33/14
3b	1/8	5/13	6/21
4	4/6	7/7	11/13

Distribution of patients by Knosp Classification
